# Single nucleotide polymorphisms affecting galantamine binding to acetylcholinesterase in Alzheimer’s disease: a structural bioinformatics study

**DOI:** 10.1007/s10822-026-00805-6

**Published:** 2026-04-09

**Authors:** Nadia Islam, Betül Akçesme

**Affiliations:** 1 Department of Genetics and Bioengineering, International University of Sarajevo, Sarajevo, Bosnia and Herzegovina; 2 Hamidiye Faculty of Medicine, Department of Medical Biology, University of Health Sciences, Üsküdar, İstanbul, Turkey

**Keywords:** Acetylcholinesterase, Galantamine, Alzheimer’s disease, SNP, Molecular dynamics simulations

## Abstract

Galantamine, an acetylcholinesterase (AChE) inhibitor used for symptomatic treatment of Alzheimer’s disease (AD), shows substantial inter-individual variability in clinical response. Missense single nucleotide polymorphisms (SNPs) within the AChE active-site gorge may modulate inhibitor recognition. In this computational study, binding residues were defined from human AChE inhibitor co-crystal structures and cross-referenced with dbSNP missense variation, followed by in-silico predictions of variant impact, evolutionary conservation and folding stability, and assessment of ligand engagement by docking and molecular dynamics (MD) with MM/GBSA binding-energy estimation. Using complexes containing galantamine (GNT) and a donepezil-like ligand (E20), 11 of 807 AChE missense variants overlapped binding-site residues, highlighting Phe294 (UniProt Phe326) and His447 (UniProt His479). ConSurf classified His447 as highly conserved, and MUpro predicted decreased folding stability for His447 substitutions. SwissDock docking indicated that His447Gln retains a plausible GNT binding pose and yielded the least favourable docking score among the tested variants, consistent with a potential reduction in binding strength. MD simulations (200 ns) of wild-type and His447Gln AChE–GNT complexes supported preserved global structural integrity of the complex over the simulated timescale, while indicating local remodelling of the GNT binding microenvironment. MM/GBSA estimates from terminal snapshots suggested a modestly less favourable theoretical binding free energy for His447Gln relative to wild-type (approximately 2.0 kcal mol⁻¹). Given that His447 is the catalytic triad histidine, such substitutions may have consequences for catalysis in addition to inhibitor binding; these in-silico findings require experimental validation using site-directed mutagenesis with kinetic and binding assays.

**Supplementary Information:**

The online version contains supplementary material available at 10.1007/s10822-026-00805-6.

## Introduction

 Alzheimer’s disease (AD) is a progressive neurodegenerative disorder characterised by cognitive decline, synaptic dysfunction, and widespread neuronal loss [1]. Therapeutic strategies have largely focused on symptomatic improvement through modulation of cholinergic neurotransmission, as well as pathways implicated in amyloid processing [2]. Acetylcholinesterase (AChE) remains a central target for symptomatic treatment, with clinically used inhibitors including galantamine (GNT), donepezil, and rivastigmine improving cholinergic signaling [3]. Beyond its canonical role in terminating cholinergic transmission at synapses by rapid hydrolysis of acetylcholine, AChE is enriched in cortical and hippocampal regions that are selectively vulnerable in AD [4], and its activity is reduced in parallel with cholinergic neuron loss [5]. The cholinergic hypothesis of AD therefore places AChE at the centre of early symptomatic treatment, and AChE inhibitors remain first line agents for mild to moderate disease. AChE has also been implicated in non-cholinergic processes relevant to AD pathophysiology [6], including promotion of amyloid β aggregation and interactions with Tau protein, which further supports interest in this enzyme as a therapeutic target beyond symptomatic benefit.

Despite the availability of approved AChE inhibitors, variability in treatment response persists. Inter-individual genetic variation in drug targets can influence protein structure, stability, and ligand recognition, potentially altering pharmacodynamic outcomes [7]. Missense single nucleotide polymorphisms (SNPs) within ligand-binding regions or adjacent structural elements may shift local interactions, affect conformational flexibility, and modify binding energetics. Understanding how such variants affect drug–target interactions is therefore relevant for target validation and may inform precision approaches [8]. Clinical studies support the relevance of genetic variability to treatment outcome in AD and other neurological disorders. For cholinesterase inhibitors, several pharmacogenetic analyses have associated variants in genes involved in cholinergic signalling or drug metabolism, such as BCHE and CYP2D6 [7, 9, 10], with differences in cognitive response and adverse effect profiles to donepezil and rivastigmine. More broadly, polymorphisms in APOE and other loci have been linked to heterogeneous responses to both symptomatic and disease modifying therapies in AD [11]. These observations indicate that common variants can meaningfully influence central nervous system drug response and motivate systematic examination of SNPs within AChE itself as a determinant of inhibitor engagement.

In-silico strategies integrating structural biology and variant annotation provide an efficient route to prioritize and characterize potentially impactful SNPs in drug targets. Databases of protein–ligand complexes enable identification of residues involved in binding, while population-scale variant repositories allow systematic mapping of missense substitutions onto these sites. Predictive tools can assess the likelihood of pathogenicity, conservation, and stability changes, and docking and MD simulations can probe the structural and energetic consequences of mutations on ligand engagement [12]. Bioinformatic and structure-based approaches have increasingly been used to connect naturally occurring variants with altered drug binding [13]. Studies across oncology, infectious disease and cardiovascular pharmacology have combined variant annotation with homology modelling, docking and MD to predict how patient derived mutations in kinases, receptors or enzymes affect inhibitor affinity and resistance. For example, in ovarian cancer, an in-silico study of the AKT1 W80R mutant combined virtual screening of approximately 12,000 flavonoids with docking, MD and 3D-QSAR modelling, and proposed taxifolin as a candidate inhibitor on the basis of high docking scores, stable MD behaviour and favourable predicted ADME properties [14]. Similarly, recent work on osimertinib induced cardiac adverse reactions has combined real world FAERS pharmacovigilance data with network pharmacology, molecular docking and MD simulations to implicate multi target interactions, including AKT1 and ALB, and to propose mechanistic links between clinical cardiotoxicity signals and stable drug–protein binding at the molecular level [15]. In several cases, such computational predictions have been consistent with experimental measurements of binding and activity, supporting their value for prioritising variants with likely functional impact. Applying a similar framework to AChE provides an opportunity to move from catalogues of SNPs towards mechanistic hypotheses about how specific substitutions might modulate GNT and donepezil binding in AD.

This study applies a multi-step computational workflow. Ligands associated with well-characterised binding in the Protein Data Bank (PDB) were selected, including (−)-galantamine (GNT) and a donepezil-like ligand (E20) used as a structural proxy to compare inhibitor engagement within the AChE gorge. Binding-site residues were identified and cross-referenced with SNP data to prioritize missense variants located within, or proximal to, ligand-interacting regions. Multiple variant effect predictors were used alongside conservation and stability analyses to rank substitutions with potential to affect protein function or drug binding. Docking was performed on wild-type and variant models to estimate the impact of substitutions on ligand affinity, with follow-up visualisation to examine changes in key interactions. To investigate mutation-driven effects on dynamics and binding energetics in greater detail, an MD simulation was carried out for the AChE–GNT complex bearing the selected substitution, followed by MM/GBSA estimation of binding free energy. Together, these analyses aim to characterize how naturally occurring missense variants in AChE may influence ligand binding and, by extension, therapeutic engagement in AD.

## Method

**Fig. 1:**
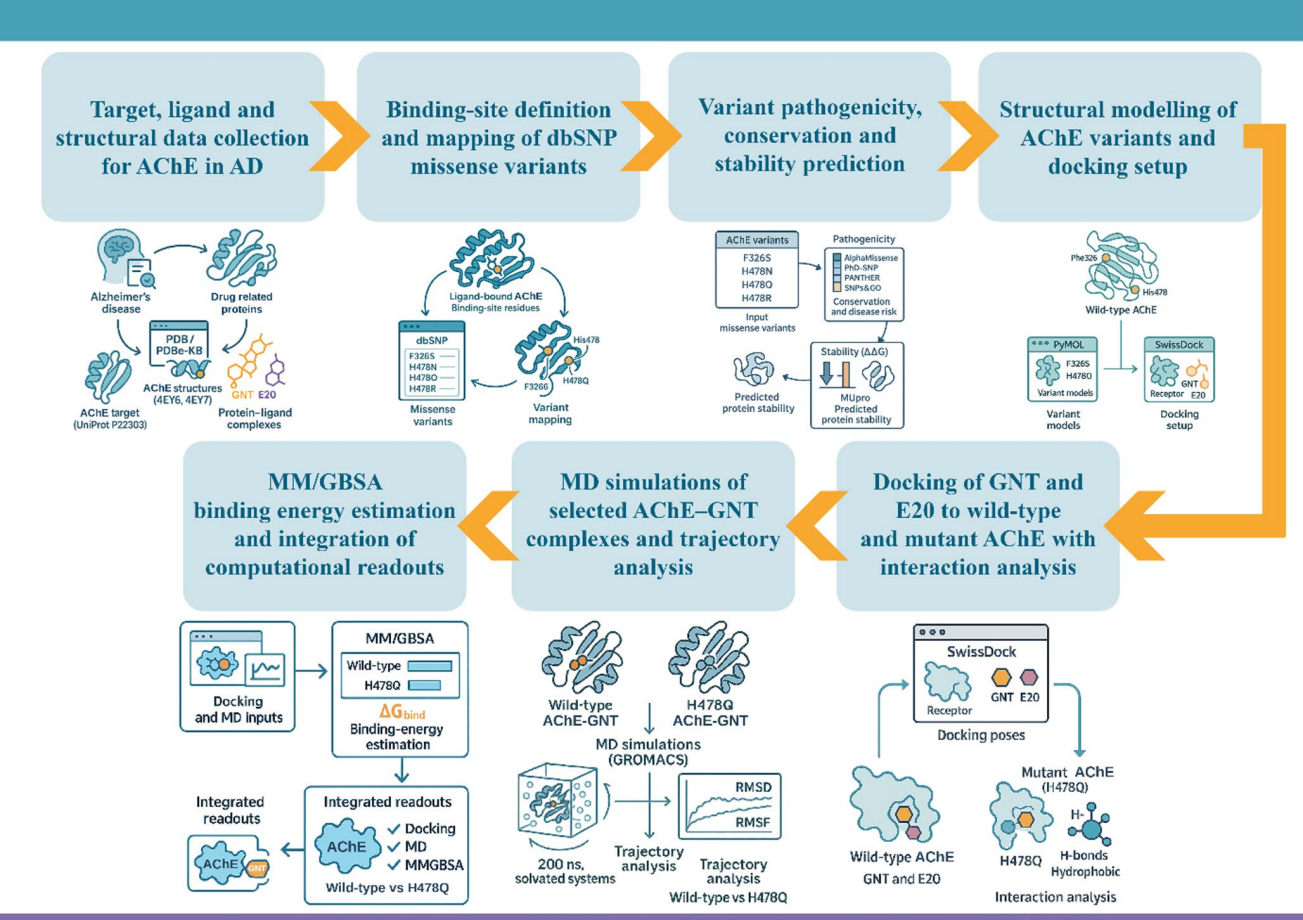
Integrated in-silico workflow for assessing the impact of AChE missense variants on GNT binding. The pipeline proceeds from target, ligand and structural data collection, through binding-site mapping and variant prioritisation, to structural modelling, docking of GNT and E20, atomistic MD simulations of selected AChE–GNT complexes, and MM/GBSA binding-energy estimation

The overall computational strategy is summarised in Fig. 1. Experimentally determined AChE structures with bound ligands were first combined with dbSNP to identify missense substitutions in the binding gorge. Variants were then prioritised using ensemble pathogenicity, conservation and stability predictors, followed by structural modelling in PyMOL and docking of GNT and E20 with AutoDock Vina within the SwissDock web server. Selected wild-type and mutant AChE–GNT complexes were subjected to explicit-solvent MD simulations, and terminal snapshots were scored by MM/GBSA, allowing comparison of docking, dynamic behaviour and estimated binding energetics for wild-type and variant systems.

### Residue numbering and sequence mapping

Structural residue indices are reported using the deposited crystal-structure numbering of PDB (https://www.rcsb.org/) 4EY6/4EY7. These structures correspond to UniProt (https://www.uniprot.org/) P22303 residues 33–574 (signal peptide omitted), such that for aligned residues UniProt position = PDB position + 32 (and PDB position = UniProt position − 32). UniProt indices are provided in brackets where relevant. A summary of the sequence correspondence and key residue conversions used throughout the study is provided in Table 1.

**Table 1:** Sequence coverage and residue-number mapping between UniProt P22303 (human AChE) and PDB 4EY6/4EY7 numbering used in this study

Item	UniProt P22303 numbering	PDB 4EY6/4EY7 numbering	Notes
Canonical protein (precursor)	1–614	–	Includes N-terminal signal peptide (1–32).
Crystallographic construct coverage	33–574	1–542	4EY6/4EY7 omit the signal peptide; PDB residue 1 corresponds to UniProt residue 33.
Index conversion (aligned residues)	UniProt position = PDB position + 32	PDB position = UniProt position − 32	Used for all structure-based analyses.
Variant site (Phe326Ser in UniProt)	Phe326	Phe294	Reported as Phe294Ser in structure-based sections.
Catalytic triad histidine	His479	His447	Catalytic triad residue.
Catalytic triad serine	Ser235	Ser203	–
Catalytic triad glutamate	Glu366	Glu334	–

### Ligand identification and binding site analysis for AD target

Based on an extensive literature search, AChE (UniProt ID: P22303) was selected for our investigation as a central cholinergic drug target in AD, where its inhibition enhances synaptic acetylcholine levels and underlies established symptomatic therapies [16]. The PDB-eKB database (https://www.ebi.ac.uk/pdbe/pdbe-kb/) was employed to identify ligands co-crystallised with this protein. Among these, the ligand GNT ((−)-galantamine) corresponds to the approved AChE inhibitor GNT, whereas E20 (1-benzyl-4-[(5,6-dimethoxy-1-indanon-2-yl)methyl]piperidine) is a donepezil-like inhibitor co-crystallised with AChE and used here as a structural proxy for the binding mode of donepezil. Binding sites for AChE were defined from these complexes and comprised amino acid residues directly involved in ligand interactions in the active-site gorge.

### Genetic variation assessment and its impact on ligand-protein interactions for AD therapeutics

Missense SNPs in AChE were retrieved from dbSNP (https://www.ncbi.nlm.nih.gov/snp/) to identify coding variation with potential to affect inhibitor engagement. Variant records were filtered to retain missense consequences in the canonical AChE protein (UniProt P22303) and deduplicated by amino-acid substitution where multiple rsIDs encoded the same protein change. To ensure consistency between sequence-based annotations and structure-based analyses, residue indices were normalised to the reference sequence used by each resource and then mapped onto the crystal structures used in this study (PDB 4EY6/4EY7). Ligand-interacting residues were defined from the co-crystal complexes described in Sect.  “Ligand identification and binding site analysis for AD target” and cross-referenced with dbSNP-derived missense substitutions to identify variants located within, or directly lining, the inhibitor-binding gorge. Variants overlapping the binding-gorge residue set were prioritised for downstream pathogenicity prediction, structural modelling (in-silico mutagenesis), docking, and MD simulation. To address ambiguity arising from different numbering schemes (for example, precursor UniProt numbering versus deposited PDB numbering), the UniProt-to-PDB residue-number conversion in Table 1 was applied throughout.

### Comprehensive computational evaluation of SNP pathogenicity in AD-related protein

An in-depth computational analysis was conducted to evaluate the pathogenic potential of SNPs in AD-related protein. Advanced bioinformatic tools such as AlphaMissense (https://github.com/Ensembl/VEP_plugins/blob/release/114/AlphaMissense.pm), PhD-SNP (https://snps.biofold.org/phd-snp/phd-snp.html), PANTHER (https://sourceforge.net/projects/pantherdb/), and SNPs&GO (https://snps.biofold.org/snps-and-go/snps-and-go.html) were utilised to predict the impact of genetic variations. dbNSFP (https://www.dbnsfp.org/) was used to aggregate additional algorithmic predictions (including SIFT, PolyPhen-2, and MutationTaster) for the same variants. Because variant consequence annotations may be reported relative to different protein reference sequences (for example, UniProt versus RefSeq isoforms), all candidate substitutions were checked for consistent protein-level descriptions, then mapped to the structural residue numbering of 4EY6/4EY7 using Table 1. Throughout the manuscript, structural interpretations (docking poses, interaction maps, MD analyses) refer to the PDB residue numbering of 4EY6/4EY7, with UniProt indices provided in brackets where relevant.

### Molecular docking and visualisation analysis of ligand-protein interactions

Molecular docking simulations were performed using the AutoDock Vina within the SwissDock web server (https://www.swissdock.ch/), which predicts ligand binding modes and estimated affinities for protein–ligand complexes [17]. Experimental AChE structures in complex with GNT and E20 were obtained from the PDB (PDB IDs: 4EY6 and 4EY7, respectively). For each complex, the bound ligand and receptor were separated into individual PDB files and the wild-type AChE–GNT and AChE–E20 systems were first redocked to the corresponding receptor as a control to verify that the docking procedure could reproduce the crystallographic binding pose.

Candidate missense variants prioritised within the binding gorge (Sect.  “Genetic variation assessment and its impact on ligand-protein interactions for AD therapeutics”) were introduced into the receptor structure using the PyMOL mutagenesis tool (https://pymol.org/). The following substitutions were modelled: Phe294Ser (UniProt Phe326Ser), and His447Asn/Gln/Arg (UniProt His479Asn/Gln/Arg). For each substitution, side-chain rotamers were selected to minimise steric clashes and maintain plausible local geometry. The resulting mutant AChE structures, without ligand, were saved as separate PDB files.

For each mutant and for the wild-type receptor, GNT and E20 were docked using SwissDock/AutoDock Vina with default settings. Docking thus provided a set of predicted complexes for wild-type and variant AChE with each ligand, enabling comparison of binding modes and estimated affinities to assess the impact of SNP-induced substitutions on interaction patterns. Docked poses were inspected and compared with the crystallographic references in PyMOL, and detailed non-covalent interactions were analysed using BIOVIA Discovery Studio Visualizer (https://discover.3ds.com/discovery-studio-visualizer-download) to identify changes in hydrogen bonding, aromatic stacking and hydrophobic contacts within the active-site gorge.

### Evaluating mutation-induced protein stability and conservation impact

To assess the impact of genetic mutations on predicted folding stability, the MUpro tool (https://mupro.proteomics.ics.uci.edu/) was employed to estimate changes in stability (ΔΔG) associated with amino acid substitutions. Additionally, ConSurf (https://consurf.tau.ac.il/consurf_index.php) analysis was conducted to determine whether these mutations occur within evolutionarily conserved regions of the proteins. Conservation analysis provides insights into the functional significance of specific residues, as mutations in highly conserved areas are more likely to affect protein function and, consequently, drug interaction effectiveness [18].

### Visualisation and structural analysis of protein mutations

To explore the structural impact of specific mutations on protein-ligand interactions, visualisation and structural analysis were conducted using the Discovery Studio Visualizer by BIOVIA (https://www.3ds.com/products/biovia/discovery-studio/visualization). Mutations were selected based on rotamer probability, binding affinity, and stability changes. The selected mutation was then analysed with ligands GNT and E20 to assess its influence within a conserved region. The wild-type and mutant protein structures were extracted from their respective PDB entries and visualised side-by-side to highlight alterations introduced by the mutations, providing insights into their potential effects on ligand binding and drug efficacy in AD treatment.

### Molecular dynamics simulations and MM/GBSA binding-energy estimation

MD simulations were performed using GROMACS 2023.5 (https://manual.gromacs.org/2023.5/download.html) for both the wild-type AChE–GNT complex and the mutant His447Gln variant (PDB 4EY6/4EY7 numbering; UniProt His479Gln). The starting structure was the top-ranked SwissDock/AutoDock Vina pose of GNT in AChE. The His447Gln substitution was introduced into the docked receptor by in-silico mutagenesis in PyMOL, and side-chain rotamers were selected to minimise steric clashes. Protein structures were then prepared by checking for missing atoms and ensuring proper organisation of each complex. Protein parameters were generated using the GROMOS 54A7 force field, which was selected to maintain a single, internally consistent force-field family with the ATB-derived galantamine parameters, thereby minimising systematic inconsistencies in non-bonded terms that can arise when combining protein and ligand parameters from different force-field families [19]. Comparative folding benchmarks indicate that additive force fields including GROMOS 54A7 can reproduce experimental folding thermodynamics and, in some cases, kinetics with good accuracy for small proteins, with performance comparable to widely used alternatives such as AMBER ff14SB and CHARMM36m [20]. The GNT topology was generated with the Automated Topology Builder (ATB), providing parameters compatible with GROMOS, and enabling a single, internally consistent force-field framework for the protein–ligand complex. Each protein–ligand complex was placed in a triclinic simulation box with at least 1.0 nm padding between solute atoms and the box boundary to minimise periodic self-interactions. Systems were solvated using the SPC water model (spc216.gro), neutralised, and brought to 0.15 M NaCl.

Energy minimisation employed the steepest-descent algorithm until the maximum force was below 1000 kJ mol − 1 nm − 1. Equilibration proceeded in two stages with position restraints on protein heavy atoms: 100 ps of NVT at 300 K using the V-rescale thermostat (τ = 0.1 ps), followed by 100 ps of NPT at 1 bar using the Parrinello–Rahman barostat (τ = 2.0 ps).

For production, three independent 200 ns simulations were performed for both wild-type and mutant systems using a 2 fs timestep under identical periodic boundary conditions. Long-range electrostatics were treated with Particle Mesh Ewald; real-space cut-offs of 1.0 nm were applied to both Coulombic and van der Waals interactions. Bonds involving hydrogen atoms were constrained with LINCS, and water geometry was constrained with SETTLE [21]. Coordinates, velocities, and energies were written every 10 ps. During GROMACS preprocessing, coordinate files may not preserve deposited PDB residue numbering and may omit chain identifiers; residue indices from MD-derived files were therefore interpreted using the UniProt↔PDB mapping defined in Table 1. Temperature, pressure, and density were monitored to confirm stable behaviour prior to and during production. All atoms were free to move, allowing the complexes to explore their conformational landscapes. Trajectory analyses used GROMACS utilities. Structural persistence and residue-level flexibility over the simulated timescale were assessed by Root Mean Square Deviation (RMSD) and Root Mean Square Fluctuation (RMSF). Representative frames and movies were prepared with PyMOL.

Post hoc binding free energy was estimated by MM/GBSA using the Tamarind.bio webserver (https://app.tamarind.bio/protein-scoring). A single terminal snapshot from each 200 ns trajectory was submitted. Binding free energy (ΔG_bind) was calculated as

$$\:\varDelta\:{G}_{bind}=\:{G}_{complex}-\:{G}_{protein}-\:{G}_{ligand},$$

with each term comprising molecular mechanical energy (bonded and non-bonded), polar solvation (GB/PB), and non-polar solvation (SASA-based). Given the single-snapshot design and the approximations inherent to MM/GBSA, these ΔG_bind values were interpreted as qualitative, hypothesis-generating estimates rather than precise binding free energies.

## Results

### Characterization of ligand binding sites in AChE

The study identified key ligand interactions for AChE, a pivotal protein in AD pathology. Using the PDB-eKB database, ligands GNT and E20 were found associated with AChE. Binding-site residues were defined from the co-crystal complexes (PDB 4EY6 and 4EY7) and are reported using PDB residue numbering (Table 1), with UniProt P22303 indices provided in brackets. The ligand-interacting residues in the active-site gorge comprised: Trp85 (UniProt Trp117), Gly120 (UniProt Gly152), Gly121 (UniProt Gly153), Tyr123 (UniProt Tyr155), Glu201 (UniProt Glu233), Ser202 (UniProt Ser234), Phe294 (UniProt Phe326), Phe296 (UniProt Phe328), Tyr336 (UniProt Tyr368), Phe337 (UniProt Phe369), and His447 (UniProt His479).

### Analysis of genetic variations in AChE

The investigation into genetic variation affecting AChE highlighted several missense SNPs with potential implications for inhibitor engagement. Initially, 807 missense SNPs were retrieved from the dbSNP database. After cross-referencing with ligand-interacting residues defined in Sect.  “Results”, 11 SNPs were identified as overlapping residues within the active-site gorge. Variant amino-acid changes are reported using UniProt P22303 numbering as typically used in dbSNP-derived protein annotations and are mapped to the corresponding PDB 4EY6/4EY7 residue indices using the conversion in Table 1 to avoid ambiguity in structural interpretation.

These prioritised variants comprised: Tyr103Phe (rs2485516674), Tyr103Asp (rs2485516689), Ser234Asn (rs2485513318), Ser324Arg (rs774522835), Ser324Asn (rs2485511101), Ser324Gly (rs2485511113), Phe326Ser (rs1790809357), and substitutions at the ligand-contacting catalytic-site histidine His479 (PDB His447), including His479Asn (rs2115990225), His479Gln (rs151107784, rs765366984), and His479Arg (rs370742086) (Table 2).

**Table 2:** Prioritised SNPs for AChE analysis (UniProt numbering; PDB mapping shown for structural analyses)

SNP ID	Mutation (UniProt P22303)	Corresponding residue (PDB 4EY6/4EY7)
rs2485516674	Tyr103Phe	Tyr71Phe
rs2485516689	Tyr103Asp	Tyr71Asp
rs2485513318	Ser234Asn	Ser202Asn
rs774522835	Ser324Arg	Ser292Arg
rs2485511101	Ser324Asn	Ser292Asn
rs2485511113	Ser324Gly	Ser292Gly
rs1790809357	Phe326Ser	Phe294Ser
rs2115990225	His479Asn	His447Asn
rs151107784	His479Gln	His447Gln
rs765366984	His479Gln	His447Gln
rs370742086	His479Arg	His447Arg

His447 (PDB numbering) is a direct-contact residue for both GNT (4EY6) and E20 (4EY7) under a 4.0 Å heavy-atom cut-off (PyMOL contact analysis) and corresponds to UniProt His479 under the UniProt↔PDB mapping in Table 1

### Computational analysis of SNP pathogenicity in AChE

Computational evaluation of variant impact (Supplementary Table S1) assessed the potential functional relevance of prioritised AChE missense substitutions using multiple predictors (Sect.  “Comprehensive computational evaluation of SNP pathogenicity in AD-related protein”). The results indicated a mixture of variants predicted to be deleterious versus tolerated. In particular, Ser234Asn (PDB Ser202Asn) and substitutions at the ligand-contacting histidine His479 (PDB His447), namely His479Asn/Gln/Arg, were classified as likely deleterious by multiple tools, whereas Tyr103Phe and Tyr103Asp (PDB Tyr71Phe/Asp) were consistently predicted to be benign or tolerated across the evaluation. Following this initial assessment, dbNSFP was used to consolidate evidence across additional algorithms and to prioritise variants for structure-based follow-up on the basis of predicted impact and their localisation within the binding gorge. This step highlighted Phe326Ser (PDB Phe294Ser; rs1790809357) and the set of histidine substitutions at His479 (PDB His447): His479Asn (rs2115990225), His479Gln (rs151107784 and rs765366984), and His479Arg (rs370742086). These variants were therefore selected for docking analysis, with His479Gln (PDB His447Gln) prioritised for MD (Table 3).

**Table 3:** Highest-impact prioritised AChE missense variants (genomic identifiers and protein substitutions)

AChE Variant ID (hg38)	AChE Variant ID (hg19)	SNP ID	Nucleotide change	Protein substitution (UniProt; PDB in brackets)
7-100893256-A-G	7-100490877-A-G	rs1790809357	A > G	Phe326Ser (Phe294Ser)
7-100892455-G-T	7-100490076-G-T	rs2115990225	G > T	His479Asn (His447Asn)
7-100892453-G-T	7-100490074-G-T	rs151107784	G > A,C, T	His479Gln (His447Gln)
7-100892651-A-C	7-100490272-A-C	rs765366984	A > C	His479Gln (His447Gln)
7-100892652-T-C	7-100490273-T-C	rs370742086	T > C	His479Arg (His447Arg)

Protein substitutions are reported in UniProt P22303 numbering as used in dbSNP-style protein annotations. Corresponding PDB 4EY6/4EY7 residue numbers are shown in brackets using the mapping in Table 1

### Molecular docking analysis of ligand-protein interactions

Wild-type AChE structures were obtained from the co-crystal complexes 4EY6 (AChE–galantamine, GNT) and 4EY7 (AChE–E20). Following prioritisation of five high-interest missense variants, mutant receptors were generated by in-silico mutagenesis in PyMOL using deposited PDB residue numbering (Table 1). Substitutions modelled comprised Phe294Ser (UniProt Phe326Ser; rs1790809357) and variants at the ligand-contacting catalytic histidine His447 (UniProt His479), namely His447Asn (rs2115990225), His447Gln (rs151107784 and rs765366984), and His447Arg (rs370742086). SwissDock/AutoDock Vina was used to dock GNT and E20 into the wild-type and mutant receptors under default settings, and docking scores were taken from the AutoDock Vina score reported in the SwissDock/AutoDock Vina output for the top-ranked pose. All values reported in Table 4 were extracted from the AutoDock Vina line for MODEL 1 in the SwissDock output files to ensure consistent scoring across wild-type and variant systems.

Wild-type redocking yielded Vina scores of − 7.62 kcal mol⁻¹ for AChE–GNT and − 11.07 kcal mol⁻¹ for AChE–E20, providing baseline values for comparison with variant receptors (Table 4). For the AChE–GNT variant set, Vina scores spanned − 7.54 to − 6.92 kcal mol⁻¹, indicating mutation-dependent variation in the docking score. Across the tested variants, His447Gln produced the least favourable docking score for GNT. For AChE–E20, docking scores for variant receptors were more favourable than those for GNT across the same substitutions.

**Table 4:** Docking analysis of wild-type and SNP-induced substitutions, rotamer probabilities and SwissDock /AutoDock Vina scores (residue numbering: PDB 4EY6/4EY7; UniProt shown in brackets)

Ligand	Protein	Mutation (PDB; UniProt)	SNP ID	Rotamer probability	Vina score (kcal mol⁻¹)
GNT	AChE	WT	–	–	−7.62
		Phe294Ser (Phe326Ser)	rs1790809357	13.30%	−7.47
		His447Asn (His479Asn)	rs2115990225	11.50%	−7.51
		His447Gln (His479Gln)	rs151107784, rs765366984	1.20%	−6.92
		His447Arg (His479Arg)	rs370742086	8.50%	−7.54
E20		WT	–	–	−11.07
		Phe294Ser (Phe326Ser)	rs1790809357	13.30%	−11.65
		His447Asn (His479Asn)	rs2115990225	11.50%	−11.65
		His447Gln (His479Gln)	rs151107784, rs765366984	1.20%	−11.50
		His447Arg (His479Arg)	rs370742086	8.50%	−11.74

### Impact of genetic variations on protein stability and conservation in AChE

The impact of selected missense substitutions on predicted folding stability was assessed using MUpro, alongside evolutionary conservation analysis using ConSurf. MUpro predicted decreased stability (negative ΔΔG) for Phe294Ser (UniProt Phe326Ser; rs1790809357) and for substitutions at the ligand-contacting catalytic-site histidine His447 (UniProt His479), including His447Asn, His447Gln, and His447Arg (Table 5). Consistent with functional importance, ConSurf classified His447 (UniProt His479) as highly conserved (grade 9). In contrast, Phe294 (UniProt Phe326) was predicted to lie in a less conserved region in our ConSurf analysis (grade 3), suggesting weaker evolutionary constraint at this position than at the catalytic histidine.

**Table 5:** MUpro-predicted folding stability changes (ΔΔG) for prioritised AChE variants (residue numbering: PDB 4EY6/4EY7; UniProt shown in brackets)

Protein	Mutation (PDB; UniProt)	SNP ID	MUpro ΔΔG
AChE	Phe294Ser (Phe326Ser)	rs1790809357	−1.993
AChE	His447Asn (His479Asn)	rs2115990225	−1.802
AChE	His447Gln (His479Gln)	rs151107784; rs765366984	−0.107
AChE	His447Arg (His479Arg)	rs370742086	−0.048

**Fig. 2:**
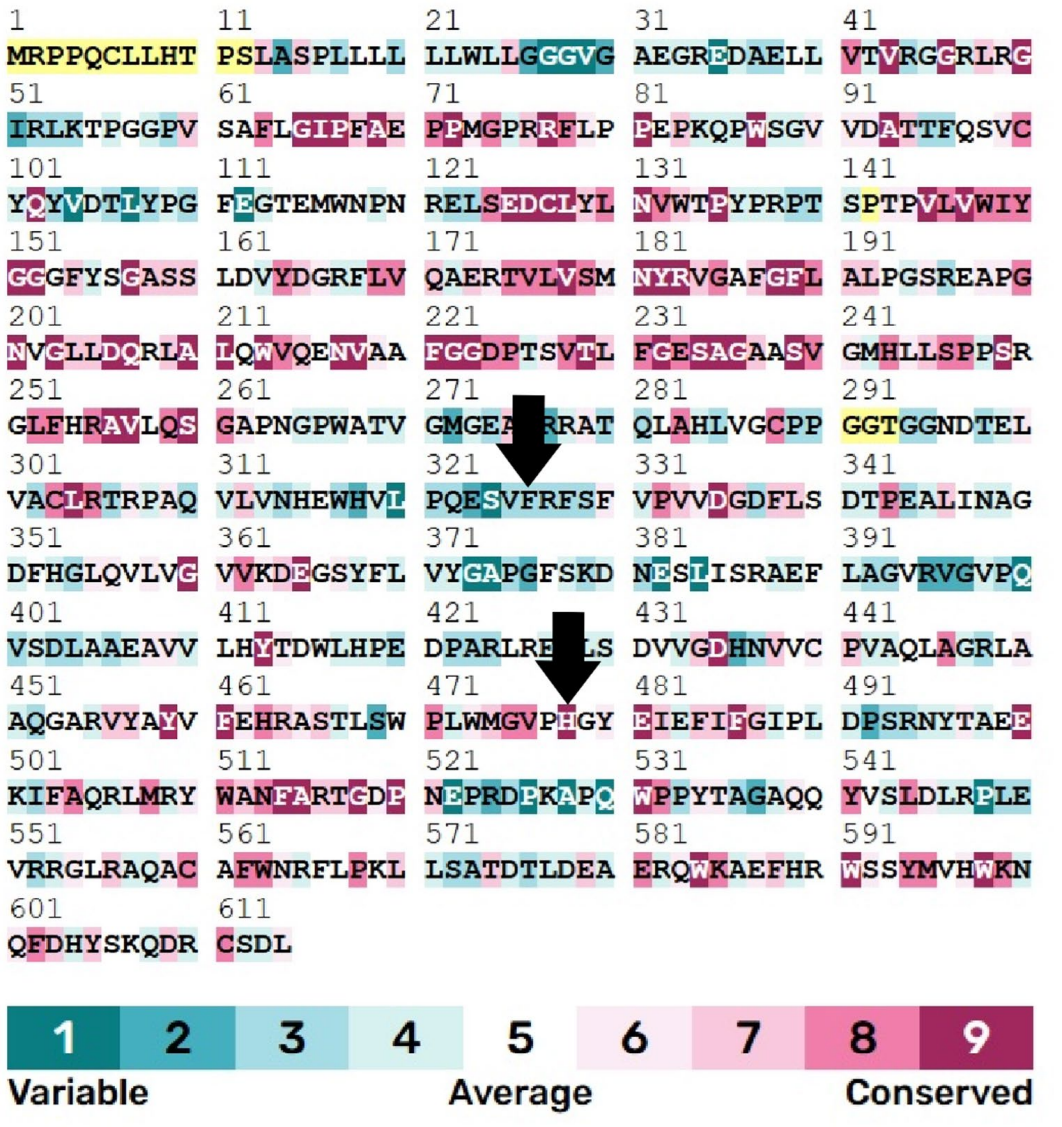
ConSurf conservation analysis for human AChE. Arrows indicate Phe326 (PDB Phe294) and His479 (PDB His447)

ConSurf mapping across human AChE (Fig. 2) supports prioritisation of His447 substitutions for structure-based follow-up, given the combination of ligand proximity (Sect.  “Results” and “Molecular docking analysis of ligand-protein interactions”) and strong evolutionary conservation at the catalytic histidine.

### Visualisation and comparative analysis of key mutations

**Fig. 3:**
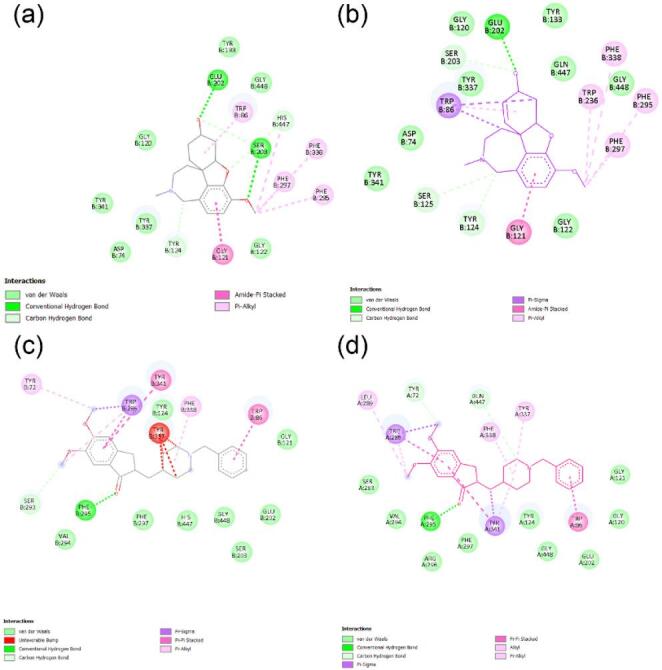
2D interaction analysis of wild-type and mutant AChE–ligand docking poses generated in BIOVIA Discovery Studio Visualizer. Panels (a–d) depict AChE complexes with ligands GNT and E20: (a) wild-type AChE–GNT showing van der Waals contacts, conventional hydrogen bonds, carbon–hydrogen bonds, amide–π stacking and π–alkyl interactions; (b) His447Gln mutant AChE–GNT exhibiting similar contacts plus additional π–sigma interactions; (c) wild-type AChE–E20 displaying van der Waals contacts, an unfavourable bump, conventional hydrogen bonds, carbon–hydrogen bonds, π–sigma, π–π stacking and π–alkyl interactions; (d) His447Gln mutant AChE–E20 retaining comparable interactions, including alkyl contacts, and lacking the unfavourable bump in the inspected pose. Colour coding: green, van der Waals; dark green, conventional hydrogen bond; light green, carbon–hydrogen bond; cyan, halogen (fluorine); orange, π–sulfur; purple, π–sigma; pink, π–alkyl; magenta, amide–π stacking; grey, unfavourable bump

For comparative visual analysis, substitutions showing distinct behaviour across rotamer plausibility, docking scores, and MUpro-predicted folding stability were examined in BIOVIA Discovery Studio Visualizer. The His447Gln variant (UniProt His479Gln; rs151107784 and rs765366984) was selected for detailed inspection because it exhibited the lowest rotamer probability in our set (1.2%), a modest predicted decrease in folding stability (MUpro ΔΔG = − 0.1067), and the least favourable docking score for GNT among the tested variants. In docking, His447Gln yielded docking scores of − 6.92 kcal mol⁻¹ with GNT and − 11.50 kcal mol⁻¹ with E20. ConSurf indicated that His447 (UniProt His479) is highly conserved (grade 9), consistent with functional constraint at this position.Comparative 2D interaction maps (Fig. 3) illustrate wild-type and His447Gln AChE complexes with GNT and E20. For GNT, the wild-type complex shows van der Waals contacts, conventional hydrogen bonds, carbon–hydrogen bonds, amide–π stacking and π–alkyl interactions. The His447Gln complex broadly retains these contacts and additionally displays π–sigma interactions, consistent with local rearrangement of the binding microenvironment rather than gross loss of ligand engagement. For E20, the wild-type complex presents van der Waals contacts, an unfavourable steric contact (“unfavourable bump”), conventional hydrogen bonds, carbon–hydrogen bonds, π–sigma, π–π stacking and π–alkyl interactions. The His447Gln complex preserves a comparable interaction pattern, including alkyl contacts, and lacks the unfavourable bump in the inspected pose. Overall, these visualisations support preserved ligand accommodation in the mutant, with mutation-associated local remodelling of interaction patterns within the gorge.

### Molecular dynamics assessment of AChE–GNT His447Gln with post hoc MM/GBSA

MD simulations were focused on the AChE–GNT His447Gln complex (UniProt His479Gln) to assess structural persistence and ligand-binding dynamics over the simulated timescale, rather than thermodynamic folding stability. The choice of variant was motivated by (i) structural proximity to the ligand, as His447 is a ligand-contacting gorge residue in both 4EY6 (GNT) and 4EY7 (E20) under a 4.0 Å heavy-atom contact criterion, (ii) high evolutionary conservation at this position (ConSurf grade 9), and (iii) prioritisation by multiple variant-effect predictors (Supplementary Table S1), whereas Tyr103 variants were consistently predicted to be benign. Among the evaluated gorge substitutions, His447Gln further combined the lowest rotamer probability (1.2%), a modest MUpro-predicted decrease in folding stability (ΔΔG = − 0.1067), and the least favourable docking score for GNT. Although Phe294Ser (UniProt Phe326Ser) was predicted to be more destabilising by MUpro, it produced a smaller change in the GNT docking score.

Systems with E20 were not taken forward to MD for two reasons. First, E20 was included primarily as a structural control and comparator, because it is a co-crystallised donepezil-like ligand that helps benchmark gorge-residue selection and assess whether docking could reproduce a plausible binding pose in an AChE inhibitor complex. Second, E20 is not an approved therapeutic agent for AD and was not treated here as a developable lead, its pharmacokinetic and safety properties were not established within this work, and its overall drug-like profile was not evaluated. Given the substantial computational cost of replicate 200 ns explicit-solvent simulations, the MD stage was therefore restricted to galantamine, a clinically used AChE inhibitor with direct translational relevance, and was used to interrogate mutation-associated changes in binding-site dynamics for an approved ligand rather than to pursue optimisation or development questions for a crystallographic tool compound.

Post hoc MM/GBSA calculations were performed for the wild-type and His447Gln complexes using terminal snapshots from the 200 ns trajectories. ΔG_bind values are reported as the mean across the three terminal snapshots (one per replica). The wild-type AChE–GNT complex yielded an estimated ΔG_bind of − 42.1 kcal mol⁻¹, whereas the His447Gln system yielded − 40.0 kcal mol⁻¹, corresponding to a modest reduction in predicted binding favourability of approximately 2.0 kcal mol⁻¹ for the variant. Given that these MM/GBSA values were derived from single terminal snapshots, they should be interpreted as qualitative, hypothesis-generating estimates rather than precise free energies.

#### Stability and flexibility of the wild-type AChE-GNT complex

RMSD analysis of the wild-type AChE–GNT complex over 200 ns (Supplementary Figure S1a) revealed rapid equilibration within the first 20 ns, followed by a gradual relaxation to a stable regime by approximately 100–120 ns. The overall complex RMSD increased from roughly 0.25 nm at the outset to a plateau in the range 0.39–0.42 nm, exhibiting only minor fluctuations thereafter. The protein backbone RMSD closely mirrored that of the entire complex, indicating that no large-scale conformational drift occurred. Meanwhile the heavy-atom RMSD of GNT remained low throughout the simulation (circa 0.06–0.10 nm), with a slight rise near 100 ns before returning to its prior range. This persistent low variability suggests that GNT remains firmly bound within the gorge and shows no inclination to dissociate.

Per-residue RMSF over the same 200 ns trajectory (Supplementary Figure S1b) demonstrated that most Cα atoms oscillated within 0.10–0.30 nm, reflecting a largely rigid protein scaffold. Several loop regions exhibited higher flexibility, with peaks around 0.45–0.55 nm and the greatest mobility observed at the solvent-exposed C-terminus (approximately 0.65–0.70 nm). In contrast, residues lining the binding gorge showed notably lower fluctuations, corroborating the stability of the pocket and the sustained binding mode of GNT. These RMSF findings are in agreement with the RMSD profile, together confirming that the wild-type AChE–GNT complex attains and maintains a stable conformation after initial equilibration.

#### Structural persistence and flexibility of the His447Gln AChE–GNT complex

Over 200 ns, the His447Gln system reached a stable regime within the first 15–20 ns (Supplementary Figure S1c). The complex RMSD rose from ~ 0.23 nm at the start to ~ 0.35 nm by ~ 15 ns and then approached a steady plateau of 0.38–0.40 nm with only small oscillations. The protein backbone RMSD tracked the complex and showed no sustained upward drift, indicating absence of large-scale structural changes. The heavy-atom RMSD of GNT increased from ~ 0.04 nm during the initial nanoseconds to ~ 0.09–0.10 nm by ~ 20 ns and remained within that range, consistent with a persistent bound pose in the gorge.

Per-residue RMSF for His447Gln (Supplementary Figure S1d) shows most Cα atoms fluctuating between 0.10 and 0.25 nm, with pronounced peaks confined to solvent-exposed loops near residues ~ 30–40, 180–200, 260–280 and 350–380, and at the C-terminus (~ 520–540) where values approach ~ 0.65 nm. In contrast, residues that shape the binding gorge remain comparatively rigid, with RMSF generally below ~ 0.20 nm.

#### Comparative dynamics of wild-type and His447Gln AChE–GNT complexes

**Fig. 4:**
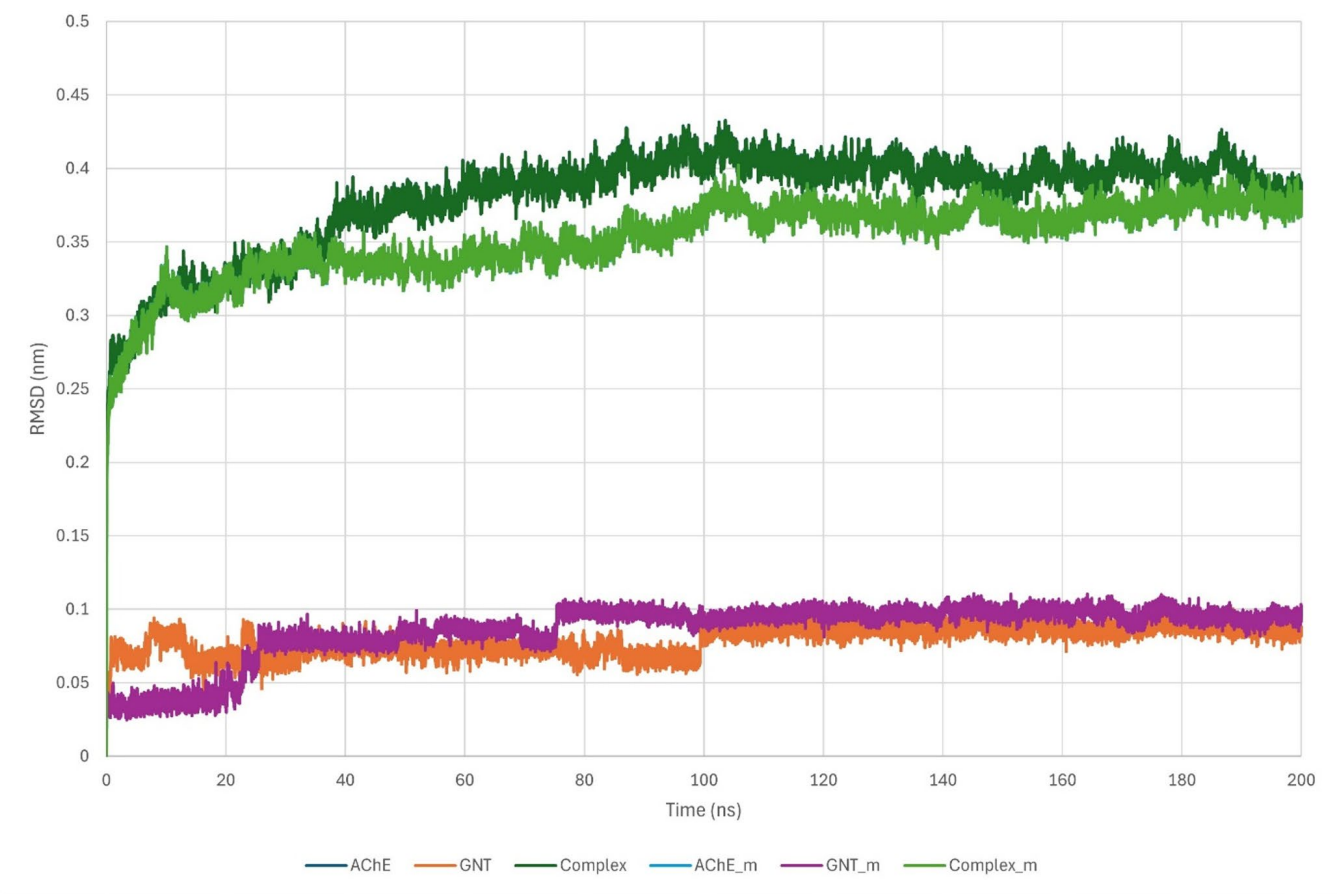
RMSD trajectories over 200 ns for wild-type and His447Gln AChE–GNT complexes, showing protein backbone, ligand and overall complex fluctuations. The mutant displays slightly reduced backbone and complex RMSD but a modest increase in ligand RMSD compared with the wild-type

Together, these data indicate that His447Gln does not compromise global structural persistence of the AChE–GNT complex on the 200 ns timescale. The substitution replaces a protonatable imidazole with a neutral amide side chain, which appears to be accommodated through local rearrangement without propagating large-scale structural changes. The plateauing complex and backbone RMSD profiles, together with low RMSF within the gorge, suggest that any MUpro-predicted decrease in folding stability is not expressed as overt conformational destabilisation within the folded complex under the simulated conditions. The modest increase in ligand RMSD is consistent with local adaptation of ligand contacts in the vicinity of residue 447 rather than weakening of the overall protein scaffold.RMSD and RMSF metrics are reported as means across three independent 200 ns replicas (± SD). Both systems reach RMSD equilibrium by ~ 20 ns and remain stable for the remainder of the 200 ns run (Fig. 4). Over the production phase, the mean complex RMSD across replicas was 0.405 ± 0.010 nm for the wild-type system and 0.385 ± 0.005 nm for His447Gln. Backbone RMSD falls from 0.280 ± 0.008 nm in the wild-type to 0.260 ± 0.005 nm in the mutant, while ligand RMSD rises from 0.070 ± 0.005 nm to 0.095 ± 0.005 nm. Minimum complex RMSD values approach 0.20 nm and maxima reach ~ 0.43 nm, reflecting primarily loop movements outside the gorge. These metrics indicate that His447Gln confers modest stabilisation to the protein and overall complex but permits slightly greater ligand mobility.

**Fig. 5:**
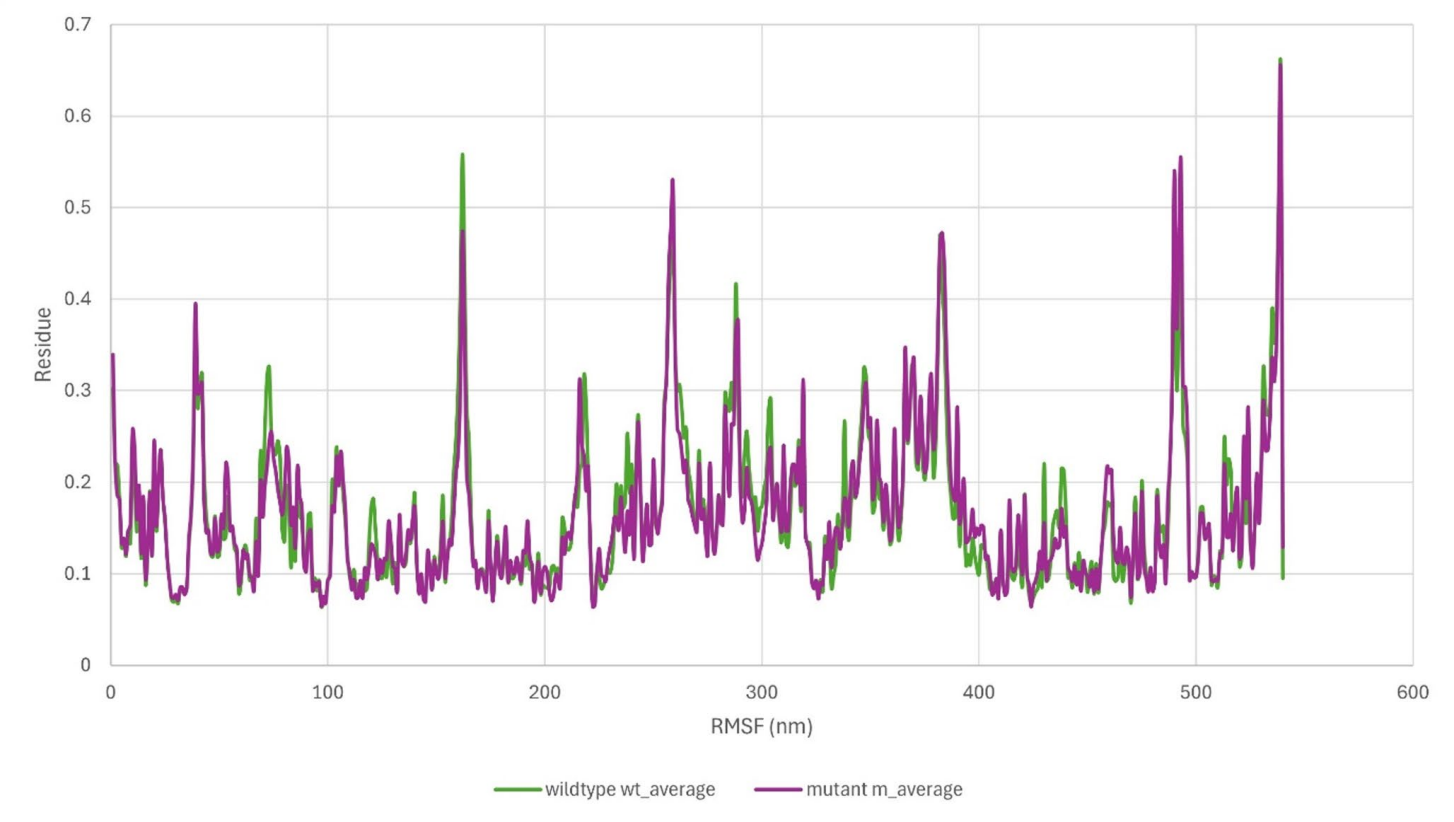
Per-residue RMSF of Cα atoms for the wild-type (green) and His447Gln mutant (purple) AChE–GNT complexes. Both systems retain low flexibility in the ligand-binding gorge, with the mutant showing a slight reduction in loop mobility around residues 180–200 and 350–380

RMSF profiles (Fig. 5) were closely similar between trajectories. Binding-gorge residues defined from the co-crystal complexes showed low fluctuations (approximately 0.08–0.15 nm), specifically Trp85 (UniProt Trp117), Gly120 (UniProt Gly152), Gly121 (UniProt Gly153), Tyr123 (UniProt Tyr155), Glu201 (UniProt Glu233), Ser202 (UniProt Ser234), Phe294 (UniProt Phe326), Phe296 (UniProt Phe328), Tyr336 (UniProt Tyr368), Phe337 (UniProt Phe369), and His447 (UniProt His479). In contrast, solvent-exposed loops (approximately residues 30–40, 180–200, 260–280 and 350–380) and the C-terminus (approximately 520–540) exhibited the largest fluctuations, peaking at approximately 0.70 nm. In the His447Gln system, loops around residues 180–200 and 350–380 showed a slight reduction in amplitude (approximately 0.02–0.03 nm), consistent with a modest local rigidification without a qualitative change in global dynamics.

**Fig. 6:**
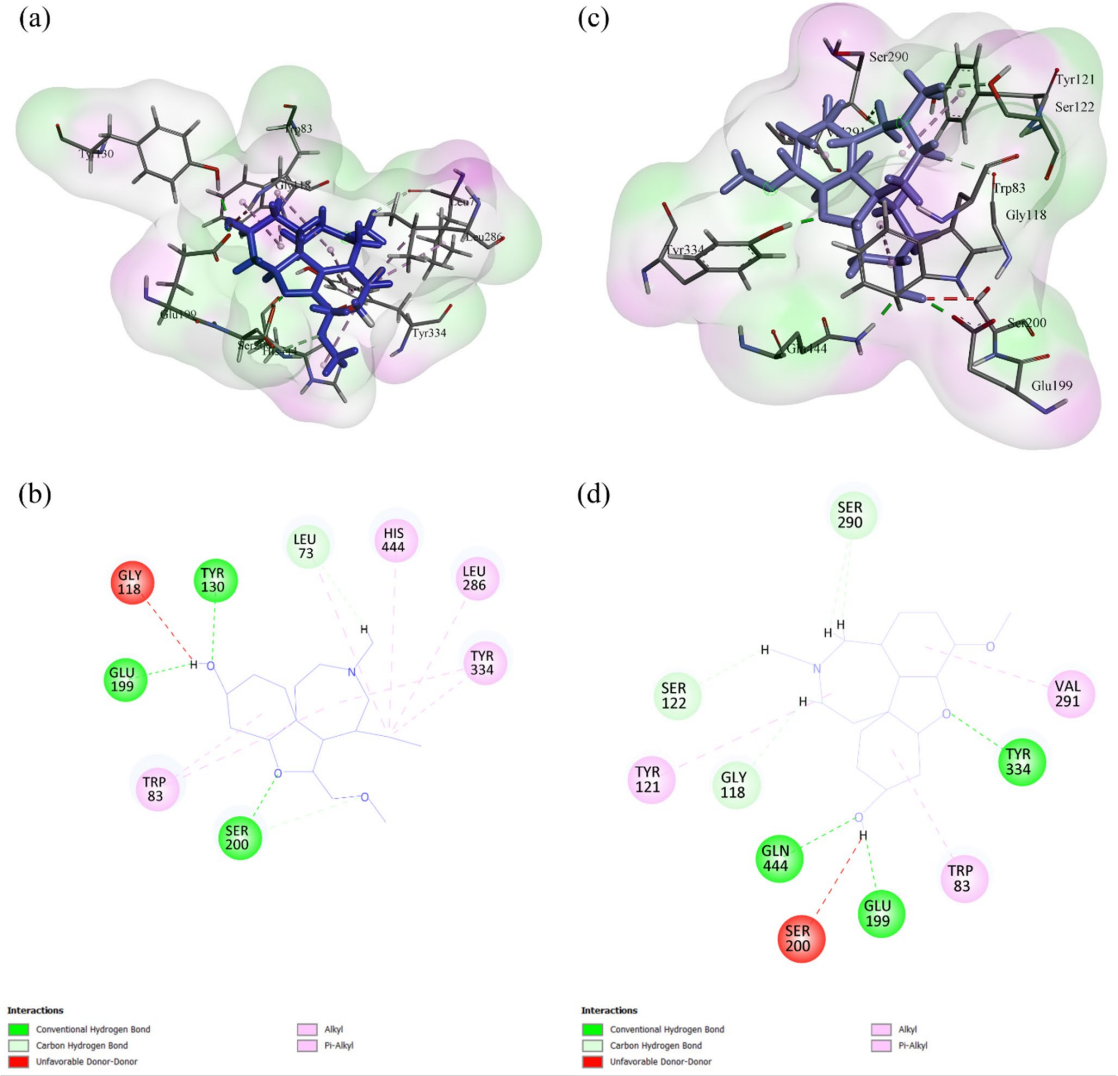
Representative 3D (**a**, **c**) and 2D (**b**, **d**) interaction maps of GNT bound to wild-type (**a**, **b**) and His447Gln mutant (**c**,**d**) AChE after 200 ns MD, generated with BIOVIA Discovery Studio. Protein surface rendering includes explicit hydrogens, and non-covalent contacts are coloured as conventional hydrogen bonds (green), carbon–hydrogen bonds (light green), and alkyl or π-alkyl interactions (purple and pink). Residue labels in these interaction diagrams follow the numbering in the analysed MD coordinate files; deposited PDB numbering is provided in Table 1 for reference

As shown in Fig. 6, GNT remained buried within the active-site gorge after 200 ns in both wild-type and His447Gln systems, and the interaction network observed in the wild-type complex was largely preserved in the variant, with local rearrangements proximal to the substituted site. These patterns are consistent with the RMSD and RMSF results, supporting maintained ligand accommodation with mutation-associated microenvironmental remodelling rather than ligand dissociation over the simulated timescale.

## Discussion

In AD, symptomatic benefit from cholinesterase inhibitors remains clinically important, yet substantial inter-individual variability in efficacy and tolerability has been repeatedly reported, consistent with a contribution from inherited polymorphisms that act through both pharmacokinetic and pharmacodynamic mechanisms [7, 9]. Within the pharmacodynamic component, coding SNPs in AChE itself were plausibly expected to modulate inhibitor engagement, particularly when substitutions occur in, or adjacent to, the active-site gorge where approved ligands such as GNT and donepezil bind [22]. Simchovitz et al. (2017) argued that both coding and non-coding SNPs affecting AChE properties and its regulation by cholinergic microRNAs (CholinomiRs), including a 3′UTR miR-608 recognition-site variant linked to elevated AChE levels and increased inflammation, could modulate susceptibility to AChE inhibition and therefore support genotype-guided, personalised approaches to AD therapy [23]. Structural data from AChE–inhibitor co-crystal complexes provide an experimentally grounded basis for defining these interaction networks, which are dominated by aromatic and hydrophobic contacts along the gorge and discrete hydrogen-bonding patterns that shape ligand positioning and microstate occupancy. Missense substitutions at gorge-lining residues can therefore be expected to produce predominantly local perturbations, such as side-chain repacking, altered hydrogen-bond geometry, and changes in local solvation, rather than global fold disruption, while still shifting binding energetics in ways that could be relevant to personalised treatment.

AChE terminates cholinergic neurotransmission by rapidly hydrolysing acetylcholine (ACh) in the synaptic cleft [24]; in AD, degeneration of basal forebrain cholinergic projections reduces ACh availability and contributes to impaired attention and memory. Pharmacological inhibition of AChE (for example by galantamine, donepezil or rivastigmine) slows ACh breakdown [25, 26], thereby increasing the residence time and effective concentration of ACh at muscarinic and nicotinic receptors, which can partially restore cholinergic signalling and improve cognition and activities of daily living in mild to moderate disease [27].

This study combined variant annotation, docking and MD simulation to examine how naturally occurring missense changes in AChE protein might influence inhibitor recognition and binding-site interaction patterns. Binding-site mapping from PDB structures, conservation analysis and in-silico pathogenicity predictors converged on a small set of candidate substitutions. Among these, His447 variants (UniProt His479) were selected because His447 is a ligand-contacting gorge residue in the co-crystal structures and is highly conserved (ConSurf grade 9), and substitutions at this position were repeatedly prioritised by multiple predictors. The prioritisation step therefore highlighted residues with plausible functional importance for inhibitor engagement and, for catalytic-site residues, potentially enzymatic activity.

AChE His447Gln (UniProt His479Gln) was then selected for detailed analysis because it lies within the ligand-binding gorge, shows the lowest rotamer probability in our set, and was associated with a modest MUpro-predicted decrease in folding stability (ΔΔG). Because His447 is the catalytic triad histidine (PDB His447; UniProt His479), substitutions at this position may affect catalysis in addition to inhibitor binding, and the present simulations do not quantify kinetic consequences. Prior mutagenesis and computational work in human AChE has shown that alterations in acyl-pocket residues adjacent to His447 can cause orders-of-magnitude losses in catalytic activity via conformational destabilisation or mispositioning of the His447 side chain, underscoring the sensitivity of catalysis to the local microenvironment around this residue [28]. In SwissDock, His447Gln retained a plausible GNT binding pose and produced the least favourable docking score among the tested variants (that is, the least negative score), consistent with a modest reduction in docking-estimated binding favourability.

Both the wild-type and His447Gln AChE–GNT systems equilibrated rapidly and remained stable over 200 ns. The mutant displayed slightly lower complex and backbone RMSD plateaus, whereas the ligand RMSD was modestly higher than in the wild-type. Per-residue RMSF profiles were closely similar between systems, with the binding gorge exhibiting uniformly low values and the largest fluctuations confined to solvent-exposed loops and the C-terminus. Taken together, the RMSD and RMSF profiles indicate that His447Gln preserves global structural integrity of the AChE–GNT complex over the simulated timescale, as expected for a single-site substitution in a structurally robust fold. The measurable effect manifests primarily as local reshaping of the binding microenvironment, reflected by a modest increase in ligand mobility and subtle rearrangements in the vicinity of residue 447 rather than collapse or large deformation of the gorge. Such local perturbations may be pharmacologically modulatory because they can shift specific contacts and microstate populations, but given the uncertainty of docking and MM/GBSA estimates and the modest effect sizes observed here, they are unlikely to account for clinical variability on their own without contributions from pharmacokinetic and regulatory factors [29].

Methodologically, the study benefited from using experimentally derived structures, a harmonised variant-prioritisation pipeline and inspection of interaction patterns alongside scores. The MD results added time-resolved information that cannot be obtained from docking alone, particularly the observation that the binding gorge remained dynamically restrained in both wild-type and mutant. A rigorous estimate would require ensemble averaging over multiple snapshots and replicate trajectories, and ideally alchemical free-energy methods or enhanced sampling focused on binding and unbinding pathways [30]. Accordingly, the reported docking scores and single-snapshot MM/GBSA values should be interpreted as qualitative and are subject to substantial methodological uncertainty.

From a broader pharmacogenetic perspective, the observation that a highly conserved binding site substitution leaves GNT engagement largely intact is compatible with clinical reports in which variability in response to cholinesterase inhibitors has more often been associated with polymorphisms in genes such as CYP2D6, CYP3A4, BCHE and APOE than with coding changes in AChE itself [31, 32]. In contrast, target coding variants are well established determinants of drug efficacy and dose requirements in other settings, such as EGFR mutations for tyrosine kinase inhibitor response in non-small cell lung cancer or VKORC1 variants in warfarin dosing [33, 34]. Our results instead indicate that His447Gln remodels the GNT binding environment locally, through subtle rearrangements within the active-site gorge, without perturbing the overall AChE fold over the simulated timescale. This supports a model in which target-site variation can modulate specific inhibitor contacts and local binding energetics rather than inducing global architectural changes, and it motivates systematic screening of additional AChE SNPs to identify substitutions that produce larger local perturbations of inhibitor binding.

Several limitations should be acknowledged at this point. First, allele frequencies for the prioritized variants were not evaluated using population databases like gnomAD. Mutations in highly conserved regions of the AChE active-site gorge, such as His447Gln, are usually very rare in the general population. Because they are so rare, these specific SNPs probably do not explain the widespread differences in how patients respond to galantamine treatment overall. However, finding these rare variants is still important for personalized medicine. For the specific patients who carry these mutations, the changes in the binding pocket could significantly affect how well the drug works.

Docking scores have known for uncertainty and do not always correlate with experimental affinities, particularly across chemotypes or when protonation and induced fit are important [35]. The MD test was confined to one mutant system for detailed analysis, with comparisons to a corresponding wild-type trajectory; broader coverage across additional variants and ligands would improve generality. E20 was used as a structural comparator for inhibitor engagement, whereas MD was restricted to galantamine to prioritise an approved therapy within available computational resources. Protonation microstates were assigned at pH 7.4, but site-specific pKa shifts in the gorge or for GNT could affect interaction patterns and would merit further assessment using constant-pH or pKa-calibrated simulations [36]. Finally, all conclusions were computational and would require experimental validation through mutagenesis, kinetic assays or biophysical measurements.

In summary, the study successfully prioritised binding-site variants in AChE and demonstrated that the substitution preserves global structural integrity of the AChE–GNT complex over the simulated timescale. As anticipated for a single-site missense change within a well-folded protein, no large-scale conformational drift was observed; backbone and complex RMSD values remained in stable plateaus and RMSF profiles showed a consistently rigid gorge, with higher mobility confined to solvent-exposed loops and the C-terminus. The mutation’s effect was instead expressed locally, with a modest increase in ligand RMSD and binding-site rearrangements compatible with altered micro-interactions within the GNT binding site rather than disruption of overall architecture. Future work should extend MD to additional AChE variants, apply ensemble-based and, where feasible, alchemical free-energy methods, evaluate alternative protonation schemes, and validate the computational predictions experimentally.

## Conclusion

We developed an integrated in-silico workflow to prioritise ligand-binding SNPs in AChE and assess their potential impact on inhibitor engagement. By combining binding-site structural information with population variant data, conservation profiling and predicted folding stability, we identified a focused set of substitutions with plausible functional relevance, particularly at Phe294 (UniProt Phe326) and the highly conserved His447 (UniProt His479). Our results suggest that a representative conserved-site variant, His447Gln (UniProt His479Gln), is unlikely to abolish overall GNT accommodation within the gorge over the simulated timescale; instead, it remodels the local binding microenvironment, with subtle shifts in interaction patterns that are consistent with preserved global structural integrity. Because His447 is the catalytic triad histidine, such substitutions may also affect enzymatic activity in addition to inhibitor binding, and experimental validation is required. This distinction is relevant for pharmacogenetics in AD, because inter-individual response may be influenced by modest, local differences in binding energetics or residence behaviour than by rare, globally disruptive variants in the target. The findings therefore support systematic, structure-guided triage of AChE coding variants to identify substitutions that locally perturb inhibitor engagement sufficiently to motivate wet-lab testing for potential effects on dose requirements, efficacy or adverse-effect liability. This approach is scalable to additional AChE variants and other cholinergic targets and can help focus experimental validation on variants most likely to be informative in genotype-aware therapeutic studies.

## Supplementary Information

Below is the link to the electronic supplementary material.

Supplementary Material 1

## Data Availability

No datasets were generated or analysed during the current study.
