# Supplementary material for: Single nucleotide polymorphisms affecting galantamine binding to acetylcholinesterase in Alzheimer’s disease: a structural bioinformatics study
Source: J Comput Aided Mol Des. 2026 Apr 9;40(1):96. doi: 10.1007/s10822-026-00805-6 (PMC13061798; doi:10.1007/s10822-026-00805-6)
Supplement: Supplementary file 1 — Supplementary Material 1 [file 10822_2026_805_MOESM1_ESM.docx]

**Supplementary Information**

**Figure S1:** MD analysis of the wild-type (a and b) and mutant H478Q AChE–GNT (c and d) complex over 200 ns. (a) RMSD of the protein backbone (blue), GNT heavy atoms (orange) and the entire complex (green) plotted against simulation time, showing initial equilibration within the first 20 ns and a stable plateau thereafter. (b) Per-residue RMSF of Cα atoms plotted against residue number, revealing reduced mobility in the ligand-binding gorge and elevated flexibility at solvent-exposed loops and the C-terminus. (c) RMSD of the protein backbone (blue), GNT heavy atoms (orange) and the entire complex (green) plotted against simulation time, showing rapid stabilisation by ~20 ns and a sustained plateau thereafter. (d) Per-residue RMSF of Cα atoms plotted against residue number, indicating preserved rigidity within the ligand-binding gorge and enhanced flexibility at solvent-exposed loops and the C-terminus.


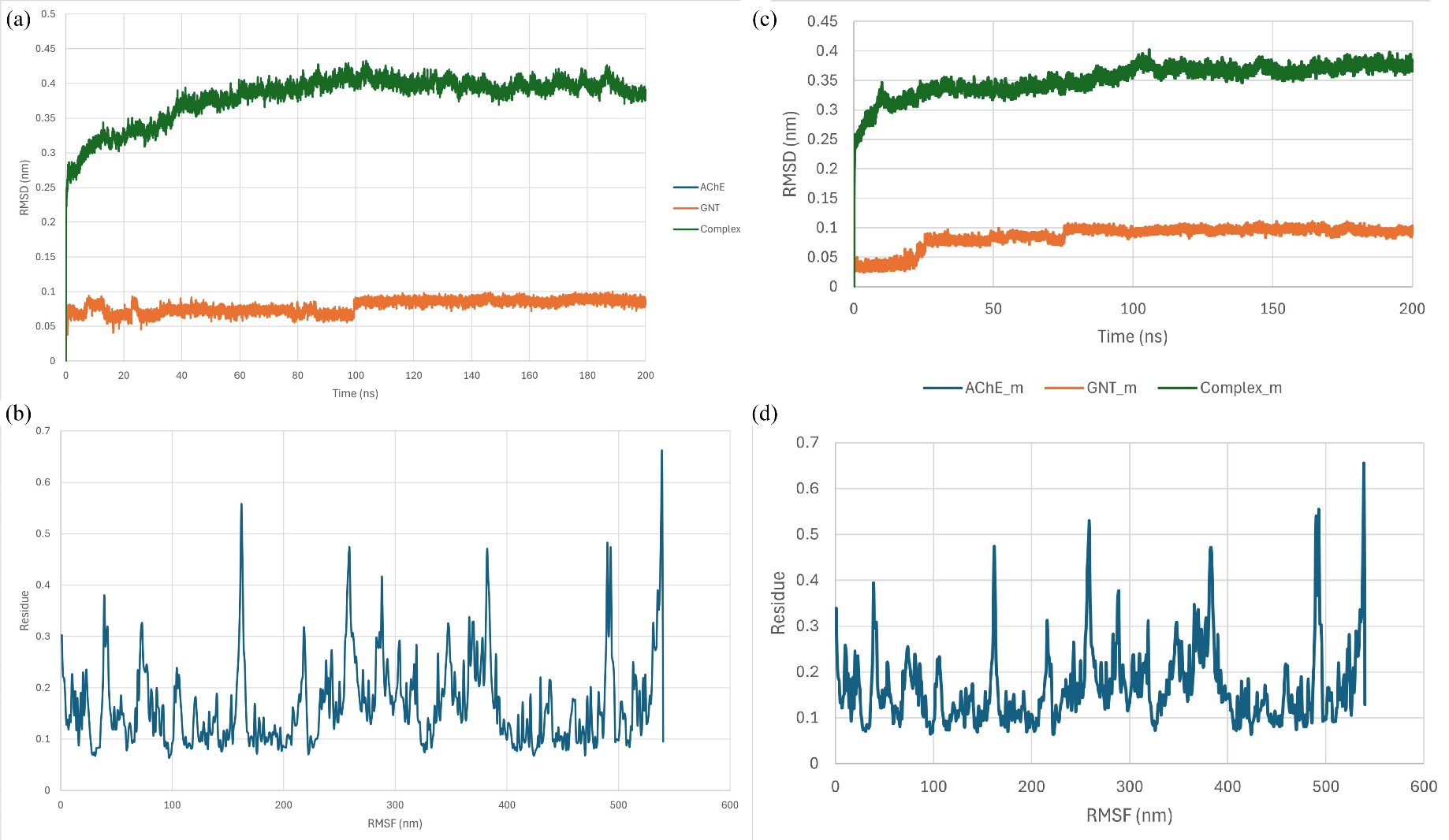


**Table S1:** Integrated in silico pathogenicity predictions for ligand binding missense variants in human acetylcholinesterase (AChE)

| **SNPs** | **Mutations** | **Drug Target** | **AlphaMissense** | **AlphaMissense Probability** | **PhD-SNP** | **PhD-SNP Probability** | **PANTHER** | **PANTHER Probability** | **SNPs&GO** | **SNPs&GO Probability** |
| --- | --- | --- | --- | --- | --- | --- | --- | --- | --- | --- |
| 2485516674 | Tyr103Phe | E20 | likely_benign | 0.088 | Neutral | 0.298 | Neutral | 0.279 | Neutral | 0.196 |
| 2485516689 | Tyr103Asp | E20 | likely_benign | 0.306 | Neutral | 0.198 | Neutral | 0.238 | Neutral | 0.094 |
| 2485513318 | Ser234Asn | GNT | likely_pathogenic | 0.978 | Disease | 0.646 | Disease | 0.957 | Disease | 0.633 |
| 774522835 | Ser324Arg | E20 | likely_pathogenic | 0.948 | Neutral | 0.276 | Unclassified | NA | Neutral | 0.08 |
| 2485511101 | Ser324Asn | E20 | ambiguous | 0.367 | Neutral | 0.121 | Unclassified | NA | Neutral | 0.035 |
| 2485511113 | Ser324Gly | E20 | likely_benign | 0.144 | Neutral | 0.052 | Unclassified | NA | Neutral | 0.017 |
| 1790809357 | Phe326Ser | GNT; E20 | likely_pathogenic | 0.976 | Neutral | 0.398 | Neutral | 0.334 | Neutral | 0.129 |
| 2115990225 | His478Asn | GNT; E20 | likely_pathogenic | 0.997 | Disease | 0.837 | Disease | 0.98 | Disease | 0.698 |
| 151107784 | His478Gln | GNT; E20 | likely_pathogenic | 0.995 | Disease | 0.762 | Disease | 0.982 | Disease | 0.679 |
| 765366984 | His478Gln | GNT; E20 | likely_pathogenic | 0.995 | Disease | 0.762 | Disease | 0.982 | Disease | 0.679 |
| 370742086 | His478Arg | GNT; E20 | likely_pathogenic | 0.987 | Disease | 0.867 | Disease | 0.974 | Disease | 0.801 |
